# Reduced production of laminin by hepatic stellate cells contributes to impairment in oval cell response to liver injury in aged mice

**DOI:** 10.18632/aging.101665

**Published:** 2018-12-04

**Authors:** Liu Qian, Hui Zhang, Yuting Gu, Dechun Li, Songbing He, Hui Wang, Yiji Cheng, Wanlin Yang, Hongshuang Yu, Xiaonan Zhao, Wei Cai, Lijun Meng, Min Jin, Yanan Wang, Yanyun Zhang

**Affiliations:** 1 Department of General Surgery, The First Affiliated Hospital of Soochow University, Institutes for Translational Medicine, Soochow University, Suzhou, China; 2 Department of General Surgery, Jiading District Central Hospital Affiliated Shanghai University of Medicine & Health Sciences, Shanghai, China; 3 Shanghai Institute of Immunology, Shanghai Jiao Tong University School of Medicine and Shanghai Institutes for Biological Sciences, Chinese Academy of Sciences, Shanghai, China; * Equal contribution

**Keywords:** oval cells, hepatic stellate cells, aging, niche, DNA-dependent protein kinase

## Abstract

Aged liver is usually impaired in response to hepatic injury. Tissue-specific stem cells participate in the repair of tissue injury. However, how oval cells (OCs) respond to injury and how the process is regulated by tissue microenvironment in aged mice have not been fully understood. In this study, taking advantage of well-established murine OC activation model, we demonstrated that OCs were less activated upon injury in aged mice and the impairment was mainly attributed to dysfunction in their niche. Through analyzing global gene expression, we found that the genes differentially expressed in damaged young and aged mouse liver tissues were predominantly those required for the formation and remodeling of extracellular matrix. As one of the most important extracellular matrix components in the OC niche, laminin was shown to promote the proliferation of OCs. Not surprisingly, laminin was downregulated with aging. Consistent with the downregulation of genes encoding DNA-dependent protein kinase (DNA-PK) proteins in aged hepatic stellate cells (HSCs), inhibition of DNA-PK also led to reduced expression of laminin in HSCs. Moreover, impairment in OC activation caused by less supporting from DNA-damaged HSCs could be rescued by laminin. This study reveals a new cellular mechanism underlying impaired OCs functionality during aging.

## Introduction

Aging has been demonstrated to be associated with a progressive and widespread impairment of cellular function, therefore, resulting in an increasing risk of developing diseases including diabetes, inflammatory disease and cancer [1]. In addition, aged people are associated with poor recovery from hepatic injury [2]. The liver possesses a remarkable capacity to regenerate after injury. However, its regenerative ability is reduced in aged liver, which significantly delays the restoration of liver function [3]. Therefore, understanding the mechanisms of declined regeneration in aged liver is critical to the development of novel treatment strategies that can block the progression of aging-related liver diseases.

There are several mechanisms responsible for liver regeneration. It has been widely known that hepatocytes can proliferate drastically to compensate for parenchymal cell loss. However, when the proliferation of hepatocytes is inhibited by drugs/toxins or when liver is severely damaged [4], the progenitor cell compartment will be activated and the liver regenerates by means of hepatic progenitor cells, also called oval cells (OCs) due to their ovoid appearance in rodents [5]. OCs originate from cells present in the so-called Canals of Hering, or from blast-like cells located next to bile duct [6]. In normal condition, OCs can hardly be observed, but upon serious liver injury, they will be activated and proliferate extensively, emerge from periportal regions and gradually migrate into central vein and hepatic lobule, and are differentiated into both hepatocytes and cholangiocytes. The proliferation and extensive accumulation of cells around central vein or portal vein during liver injury are regarded as ductular reaction [7,8]. Ductular reaction also occurs in human chronic liver diseases, such as virus hepatitis, fulminant liver hepatitis, cholestatic obstruction, cirrhosis and liver cancer [4,9]. 3,5-diethoxycarbonyl-1,4-dihydrocollidine (DDC) diet and choline deficient ethionine-supplemented diet models are widely used to induce OC response in rodents [10]. Although great progresses have been made in the understanding of the factors that regulate of OC proliferation, the changes in OC response and their regulation by microenvironment upon liver injury during aging have not been fully characterized.

The microenvironment where the liver progenitor cell reside is called OC niche, which comprises the extracellular matrix (ECM), parenchymal and non-parenchymal resident liver cells, infiltrating inflammatory cells and a great variety of growth-regulating factors. The hepatic stellate cells (HSCs) are the major resident non-parenchymal liver cells and reside in the space of Disse. Upon activated by injury, HSCs transdifferentiate into myofibroblasts, which are marked by the expression of α-smooth muscle actin (α-SMA). The activated HSCs produce ECM components including laminin and release specific cytokines or signals to regulate proliferation and differentiation of hepatocytes [11,12]. Increasing evidence has showed that HSCs are closely associated with OCs during OC growth and ductile formation in animal models [13]. OC activation and expansion are attenuated in HSC-depleted rodent liver injury model [11,14], suggesting that HSCs are positive regulator of OC proliferation. Interestingly, senescence of HSCs limits liver fibrosis, and senescent HSCs exhibit reduced secretion of ECM components and enhanced secretion of ECM-degrading enzymes [15]. However, the effect of senescent HSCs on OC response under physiological aging condition remains poorly understood.

In this study, we studied the effect of senescent microenvironment on OC activation in mice. We found that OC activation was compromised with aging, which was mainly due to dysfunction of the senescent niche. Among the niche components, laminin was demonstrated to support the proliferation of OCs. Meanwhile, laminin was downregulated with aging. Interestingly, expression of laminin in HSCs depends on DNA-dependent protein kinase (DNA-PK). Our study reveals a new cellular mechanism underlying impaired OC functionality during aging.

## RESULTS

### OC activation is impaired in aged mice

The appearance of livers in aged mice is similar to that in young mice except for occasional presence of tumor-like nodules on the liver lobes in some aged mice (Supplementary Figure S1A). The liver and body weight ratio showed no difference, while levels of serum alanine aminotransferase were comparable and total bilirubin level was higher in the aged group (Supplementary Figure S1B, C). Expression of age-related genes, such as *Trp53*, *Cdkn1a*, and *Cdkn2a*, and the corresponding proteins, p53, p21, and p16, were increased in aged liver tissues (Supplementary Figure S1D, E).

**Figure 1:**
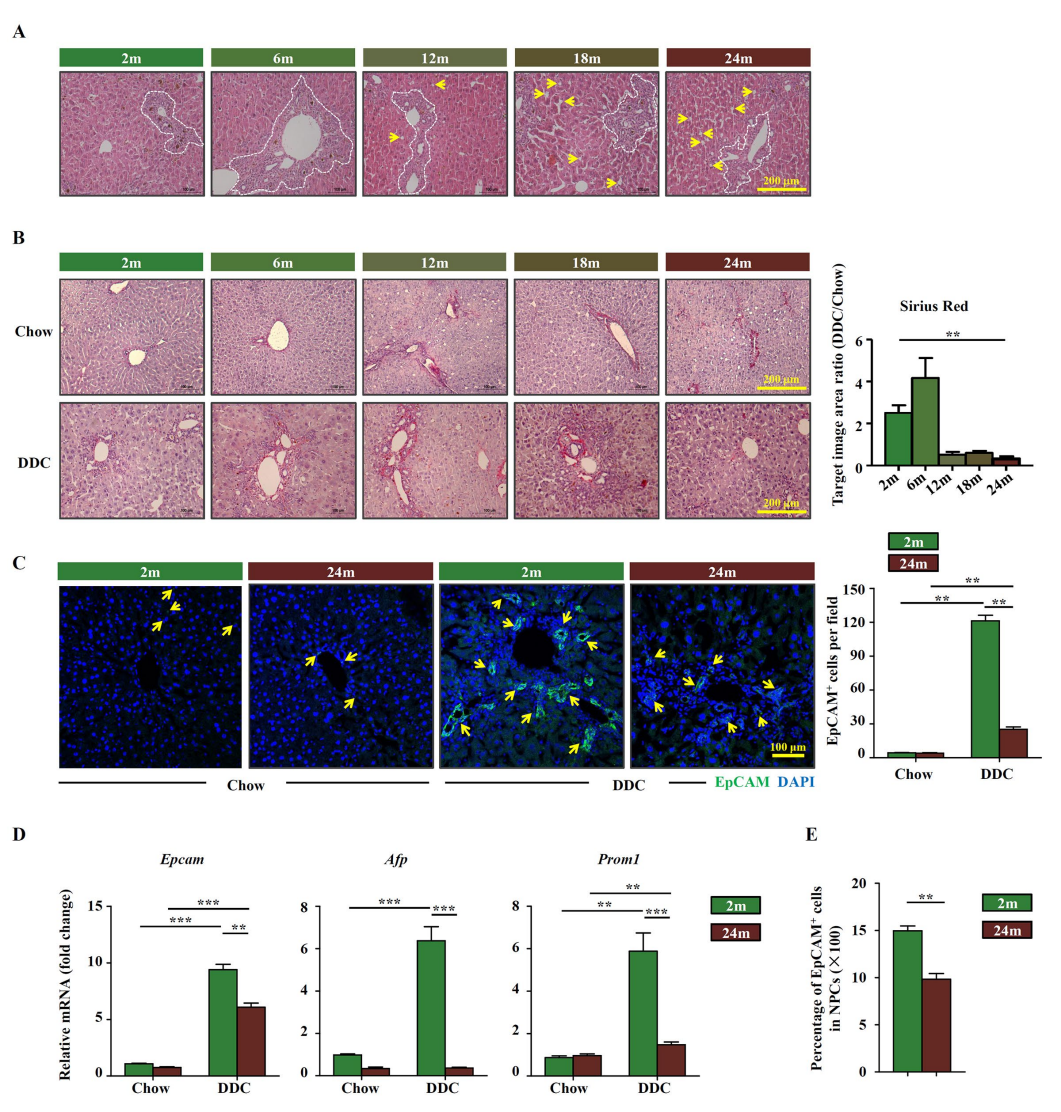
**OC activation is impaired in aged mice.** (**A**) H&E staining of DDC-fed mouse liver with the age of 2m, 6m, 12m, 18m and 24m (Scale bar=200 μm). The ductular reaction was shown in the white dashed lines outlined regions. Arrows indicated the hepatocyte steatosis. (**B**) Sirius red staining of the chow (Chow) and DDC groups (DDC) with the age of 2m, 6m, 12m, 18m and 24m (Scale bar=200 μm). The ratio of Sirius red staining area of DDC and chow groups was quantified (n=6, ** p < 0.01). Young (2m) and aged (24m) mice were fed with normal/DDC diet for 3 weeks. (**C**) Immunofluorescence staining for EpCAM^+^ cells (green) in DDC-fed (DDC) versus chow controls (Chow). Quantification of EpCAM^+^ cells was shown (n=6, ** p < 0.01). (**D**) Quantitative Real-time PCR analysis of *Epcam*, *Afp* and *Prom1* in young and aged DDC-fed mice liver (n=9, ** p < 0.01, *** p < 0.001). (**E**) EpCAM^+^ cells and NPCs were isolated from whole liver of DDC-fed young and aged mice, the ratio of EpCAM^+^ cells in NPCs was quantified (n=6, ** p < 0.01).

To determine the effects of aging on OC activation, DDC diet was used to induce liver damage in young and aged mice, respectively. Ductular reaction can be detected in different age groups but the pathological change of liver was more obvious in aged group, such as hepatocyte steatosis (Figure 1A). More collagen was deposited within ductular reaction region in young DDC-fed mice than in aged groups (Figure 1B). EpCAM staining, which reflects OC activation, was hardly detectedin both young and aged mice fed with chow diet. When fed with DDC diet, EpCAM signals were easily detected in young mice, but less so in aged mice (Figure 1C). The expression of commonly used marker genes for OCs, *Epcam*, *Afp* and *Prom1*, were also found to be increased in young DDC-fed mice while very low level in aged DDC-fed mice (Figure 1D). When OCs (EpCAM^+^ cells) were isolated from the whole liver and quantified, the percentage of OCs within the nonparenchymal cells (NPCs) was found to be lower in aged DDC-fed mice than that in young DDC-fed mice (Figure 1E). These results suggest that OC activation in response to liver injury is compro-mised in aged mice when compared to young mice.

### The altered microenvironment in aged mice affects the activation of OCs

**Figure 2:**
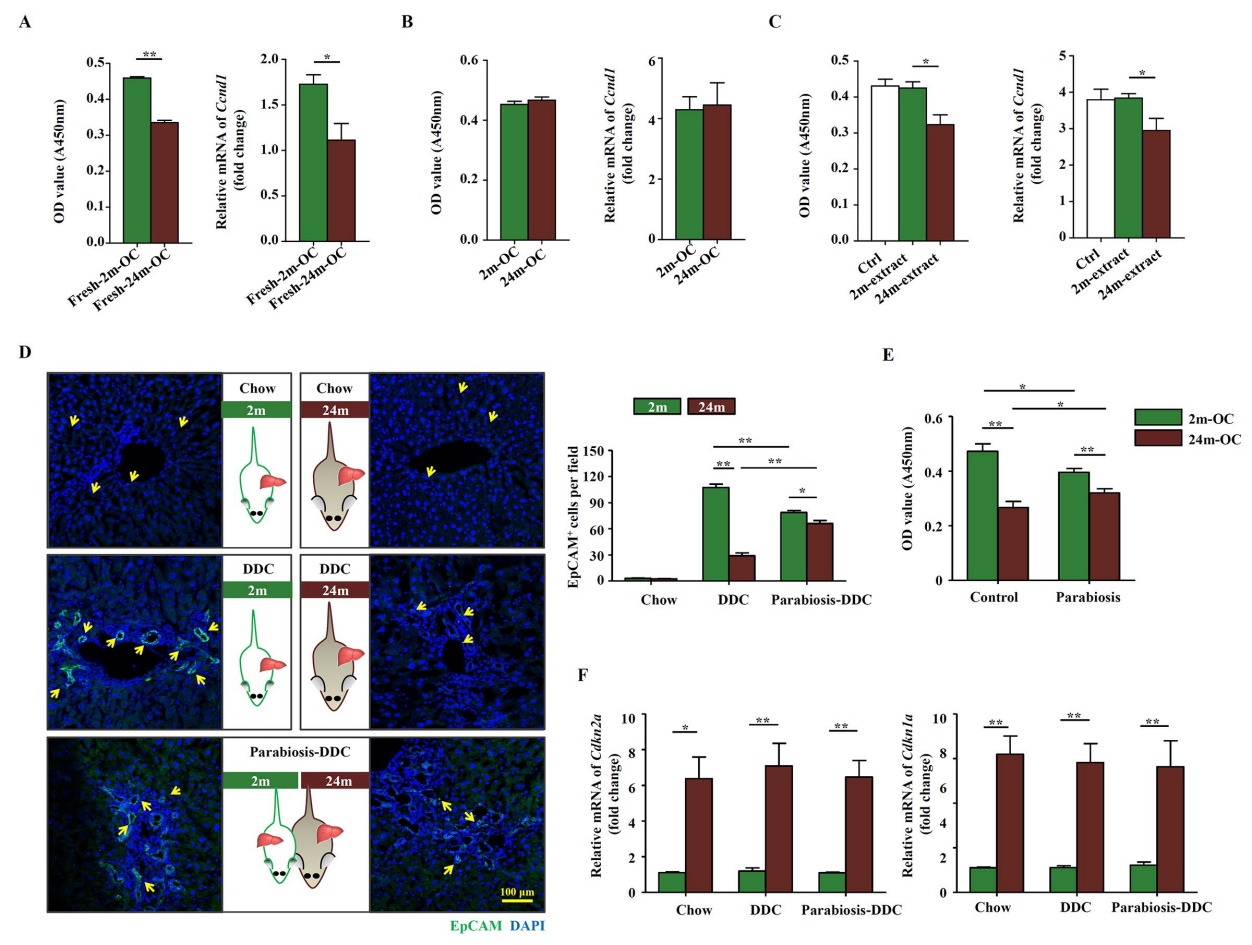
**The altered microenvironment in aged mice affects the activation of OCs.** Young (2m) and aged (24m) mice were fed with DDC diet for 3 weeks. (**A**) Freshly isolated OCs from young (Fresh-2m-OC) and aged (Fresh-24m-OC) DDC-fed mice were cultured on type I collagen coated 96-well plates, 3 days later, CCK-8 test was performed (left panel, n=6, ** p < 0.01). Quantitative Real-time PCR analysis of *Ccnd1* in 2m-OC and 24m-OC (right panel, n=6, * p < 0.05). (**B**) Freshly isolated OCs were passaged for 6 times, then CCK-8 test (left panel, n=6) and quantitative Real-time PCR analysis of *Ccnd1* (right panel, n=6) were performed. (**C**) Freshly isolated OCs were passaged for 6 times, then were cultured in normal medium (Ctrl), with liver extract from young mice (2m-extract) and with liver extract from aged mice (24m-extract). CCK-8 test (left panel, n=6, * p < 0.05) and quantitative Real-time PCR analysis of *Ccnd1* (right panel, n=6, * p < 0.05) were performed. (**D**) Young and aged mice were joined in parabiotic pairs for 3 weeks with DDC diet. Immunofluorescence staining for EpCAM^+^ cells (green) was performed. Quantification of EpCAM^+^ cells was shown (n=6, * p < 0.05, ** p < 0.01). (**E**) CCK-8 test was performed in the OCs freshly isolated from the parabiotic pair (Parabiosis) or individuals as controls (Control) (n=6, * p < 0.05, ** p < 0.01). (**F**) Young and aged mice were joined in parabiotic pairs and kept for 3 weeks under DDC diet. The transcript levels of *Cdkn2a*, and *Cdkn1a* in the liver tissues were measured by quantitative real-time PCR (n=5, * p < 0.05, ** p < 0.01).

We next explored whether the effect of aging on the activation of OCs was intrinsic or extrinsic. The proliferation potential of OCs freshly isolated from young mice fed with DDC diet was higher than that from aged mice, which was consistent with higher level of *Ccnd1*, a critical cell cycle regulator [16], in young mice (Figure 2A). No significant difference was detected between young and aged mice in the expression of OC markers including EpCAM, Sca-1, CD44, CD49f, CD45, c-kit, and CD34 (Supplementary Figure S2A). On the other hand, the expression of senescence associated genes, such as p16, p21, p53 and γ-H2AX, shared no difference between young and aged mice-derived OCs (Supplementary Figure S2B). Interestingly, when OCs isolated from young and aged mice were cultured in the same condition for several passages, the difference in proliferation rate disappeared (Figure 2B). Since some growth factors or cytokines may have impacts on the proliferation of OCs, we added insulin growth factor (IGF)-1, growth hormone (GH), Wnt3a, TGF-β and growth differentiation factor (GDF) 11 into the OC culture medium separately and cultured for 24 hours. The CCK-8 results showed that IGF-1, GH, GDF11 and Wnt3a had promotional effects on OC proliferation, while TGF-β had no effect (Supplementary Figure S3). Furthermore, when the OCs were treated with extract from aged livers, their proliferation was decreased, whereas the extract from young livers had no such effect (Figure 2C). We then generated heterochronic parabiosis using young and aged mice and subjected them to DDC diet. During the healing process after surgery, capillary anastomoses between two vascular systems allow for the exchange of circulating components. As expected, the activation of OCs in the young parabiotic partner was impeded, while that in the aged parabiotic partner was enhanced (Figure 2D). OCs isolated from parabiotic partners showed a similar pattern in their proliferation, a diminished difference between the two groups (Figure 2E). On the other hand, the expression of *Cdkn2a* and *Cdkn1a* showed no difference between parabiotic partners with their control individuals (Figure 2F). These results demonstrate that the decrease of OC activation in aged mice is most likely to be due to cell extrinsic factors. In other words, the niche probably plays a critical role in OC activation.

### Laminin supports OC proliferation via integrin signaling pathway

**Figure 3:**
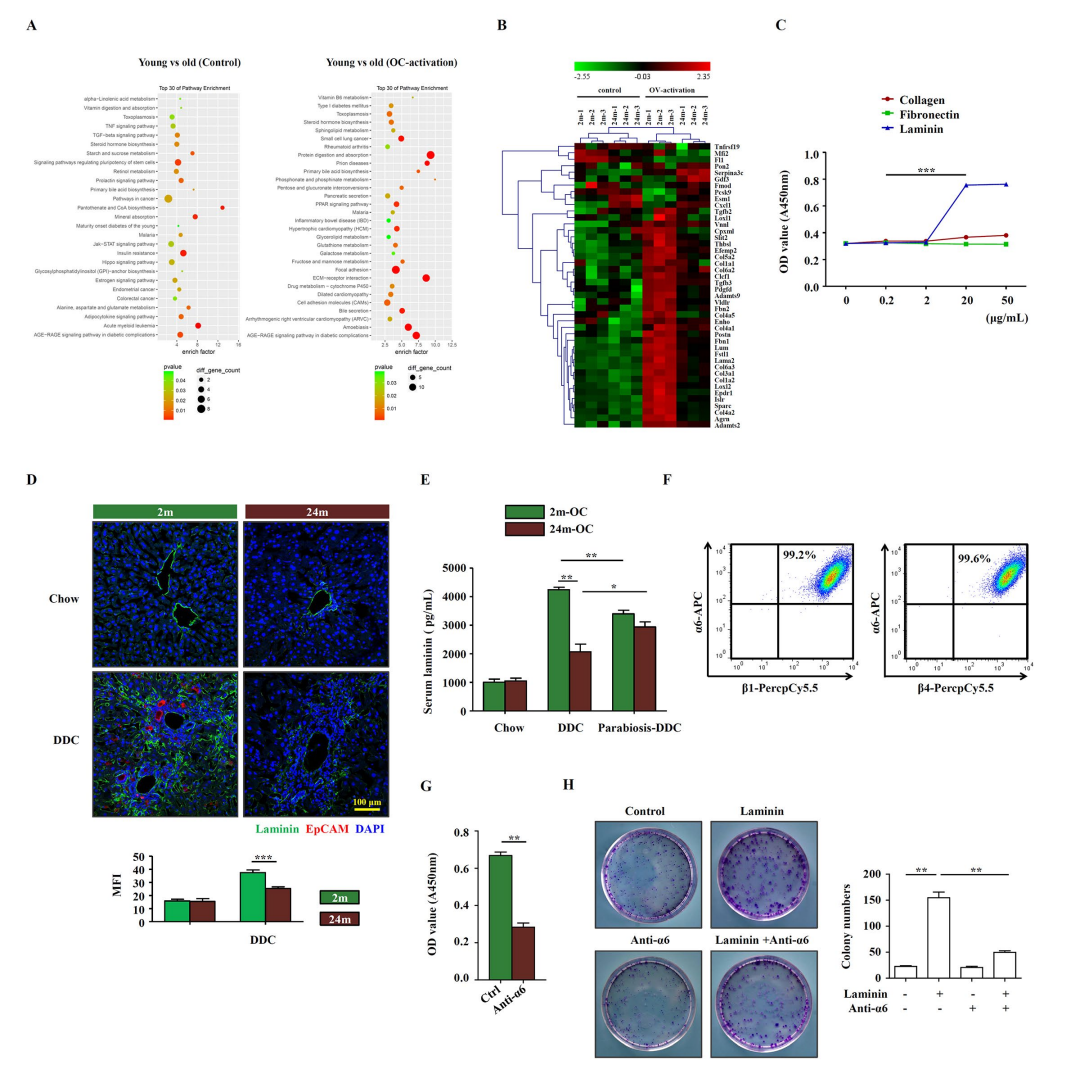
**Laminin supports OC proliferation via integrin signaling pathway.** (**A**) Microarray analysis of young and aged control/OC-activated mice liver was performed. Result of pathway enrichment analysis was shown. (**B**) Heatmap of 44 genes with significant expression difference (fold change > 5, p < 0.01) between young (2m) and aged (24m) control/OC-activated mice liver (3 samples for each group). (**C**) Different dose of collagen, fibronectin and laminin were added to the OC culture medium and the proliferation of OCs was analyzed by CCK-8 (versus 0.2 mg/mL, n=5, *** p < 0.001). (**D**) Immunofluorescence staining for laminin (green) and EpCAM (red) in DDC-fed (DDC) versus chow controls (Chow). MFI of laminin was quantified (n=6, *** p < 0.001). (**E**) Young and aged mice were joined in parabiotic pairs and kept for 3 weeks under DDC diet. The level of serum laminin was detected by ELISA (n=5, * p < 0.05, ** p < 0.01). (**F**) Flow cytometry showed the expression of α6, β1 and β4 integrins on OCs. (**G**) CCK-8 test showed that the proliferation of OCs treated with anti-α6 integrin antibody (n=6, ** p < 0.01). (**H**) Colony-forming assay of freshly isolated EpCAM^+^ cells from DDC diet with/without laminin (20 μg/mL) and anti-α6 integrin antibody (2 μg/mL). Pictures were representative wells of each condition. Colony number was quantified (n=6, ** p < 0.01).

To elucidate the mechanisms underlying the niche-regulated activation of OCs, we performed microarray analysis to identify the differentially expressed genes in the young and aged liver tissues. Pathway analysis of the molecular signature revealed that ECM-receptor interaction was one of the most noticeable pathways that were altered in the liver of aged mice, when compared to young mice (Figure 3A, B). Then, we confirmed the mRNA expression of ECM-related molecular, such as collagen, laminin, fibrillin and well-known regulatory factor TGF-β by quantitative real-time PCR, the results showed that the expression of these molecular were much lower expressed in the liver tissues of aged DDC mice (Supplementary Figure S4). Interestingly, when collagen, fibronectin and laminin (main ECM components within liver) were added respectively into the culture medium for OCs, laminin, but not collagen or fibronectin, could promote the proliferation of OCs, and it did so in a dose dependent manner (Figure 3C). This corresponds to the finding that less laminin was detected in the aged mice than in young mice on DDC diet (Figure 3D). Meanwhile, we found that in parabiotic pairs, serum laminin level increased in aged partner, while decreased in young partner compared with unpaired ones (Figure 3E). α6 integrin was reported to be the main receptor for laminin in the liver [17]. We therefore examined α6 integrin and found that α6β1 and α6β4 were abundantly expressed on OCs (Figure 3F), indicating that laminin could have functional impact on OCs through binding to α6 integrin. Importantly, when α6 integrin neutralizing antibody was added into OC culture, the proliferation of OCs was decreased (Figure 3G), and clonogenic assay showed a similar trend (Figure 3H). Thus, these data suggest that laminin in liver microenvironment may support the activation of OCs via integrin signaling pathway.

### HSCs participate in the remodeling of OC niche by producing laminin

**Figure 4:**
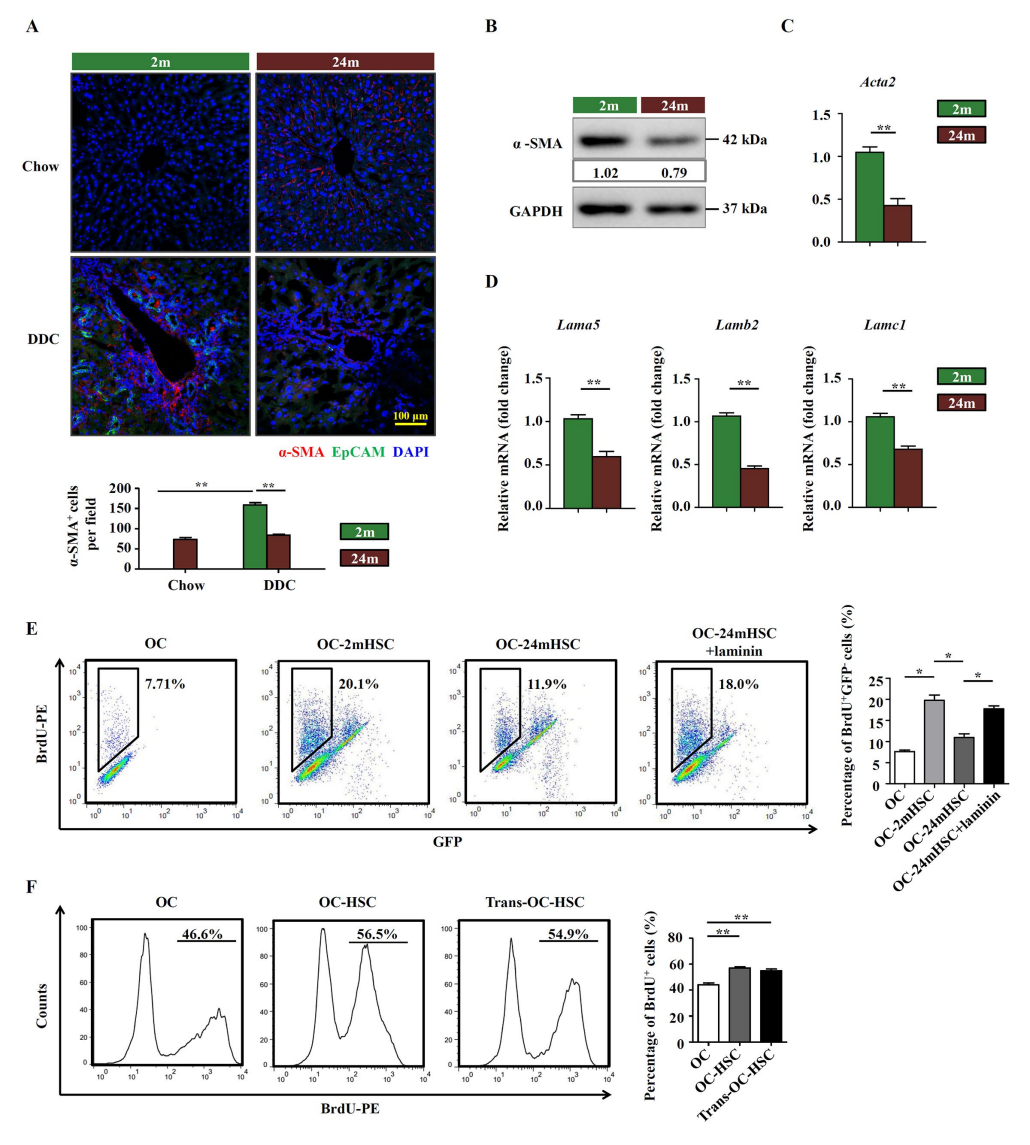
**HSCs participate in the remodeling of OC niche by producing laminin.** (**A**) Immunofluorescence staining for α-SMA^+^ (red) and EpCAM^+^ (green) cells in DDC-fed (DDC) versus chow controls (Chow) of young (2m) and aged (24m) mice. Quantification of α-SMA^+^ cells was shown (n=6, ** p < 0.01). (**B**) Western blot analysis of α-SMA in HSCs freshly isolated from young (2m) and aged (24m) mice with DDC diet. (**C**) Quantitative Real-time PCR analysis of *Acta2* in HSCs freshly isolated from young (2m) and aged (24m) mice with DDC diet (n=6, ** p < 0.01). (**D**) Quantitative Real-time PCR analysis of different laminin isotypes in HSCs freshly isolated from young and aged mice with DDC diet (n=6, ** p < 0.01). (**E**) BrdU analysis of OCs co-cultured with primarily isolated HSCs from young (OC-2mHSC) and aged (OC-24mHSC) GFP transgenic mice while in one group laminin was added (OC-24mHSC+laminin) (n=4, * p < 0.05). (**F**) BrdU analysis of OCs co-cultured with HSCs in a cell-cell contact manner (OC-HSC) or with a transwell system (trans-OC-HSC) (n=5, ** p < 0.01).

Since activated HSCs are the main producer of ECM in the liver, we speculated that HSCs may regulate OC niche via the production of laminin. Staining of α-SMA, a commonly used marker that identifies activated HSCs, indicated that significant induction of α-SMA was only detected in young DDC-fed mice (Figure 4A). The activated HSCs were located in the lobes, some surrounding EpCAM^+^ OCs (Figure 4A). Consistent with the result *in vivo*, the expression level of α-SMA was also found to be lower in isolated HSCs from aged group than in those from young mice (Figure 4B, C). As expected, the mRNA levels of *Lama5*, *Lamb2*, and *Lamc1*, the isoforms of laminin that are mainly expressed in liver, were lower in HSCs isolated from aged DDC-fed mice (Figure 4D). We also checked whether HSCs could produce laminin within the liver under treatment of DDC. The confocal results showed many the co-localization of α-SMA and laminin in DDC-fed mice liver, especially in young DDC mice (Supplementary Figure S5). To explore the effect of HSCs on OC activation, we co-cultured OCs with HSCs isolated from young and aged mice with DDC diet, respectively and found that OCs proliferated faster in the presence of HSCs. However, HSCs from aged mice were much less effective in promoting OC proliferation than those from young mice (Figure 4E). Addition of laminin to the co-culture system compensated for the incompetence of HSCs from aged mice. Transwell culture indicated that cell-cell contact was not required for the proliferation-promoting effect of HSCs (Figure 4F). Collectively, our findings indicate that HSCs participate in the remodeling of OC niche by producing laminin and their activation is impaired in aged mice, leading to declined proliferation of OCs in response to liver damage.

### Aged HSCs provide less support to OCs

**Figure 5:**
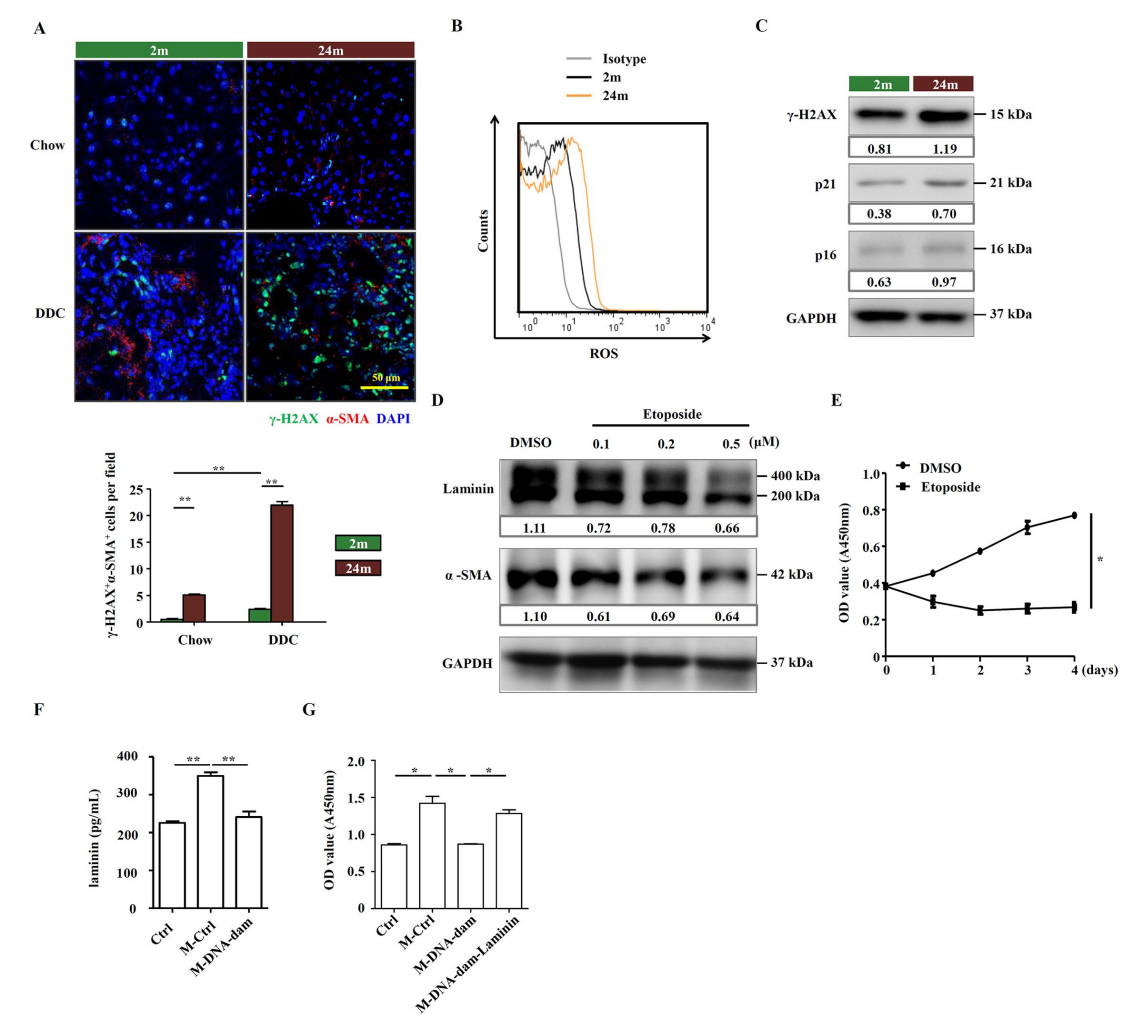
**Aged HSCs provide less support to OCs.** (**A**) Immunofluorescence staining for γ-H2AX^+^ (green) and α-SMA^+^ (red) cells in DDC-fed (DDC) versus chow (Chow) of young (2m) and aged (24m) mice. Quantification of γ-H2AX^+^α-SMA^+^ cells was shown (n=6, ** p < 0.01). (**B**) ROS levels were analyzed in HSCs freshly isolated from young (2m) and aged (24m) mice with DDC diet. (**C**) Western blot analysis of indicated proteins in HSCs freshly isolated from young (2m) and aged (24m) mice with DDC diet. (**D**) Western blot analysis of indicated proteins in JS1 cells pretreated with different doses of etoposide for 24 hours. (**E**) CCK-8 test showed the proliferation of JS1 cells treated with etoposide (0.5 μM; n=5, * p < 0.05). (**F**) The level of laminin in normal culture condition (Ctrl), conditional medium of HSC supernatant (M-Ctrl), and conditional medium of etoposide pretreated HSC supernatant (M-DNA-dam) was measured by ELISA (n=5, ** p < 0.01). (**G**) CCK-8 test shows that proliferation of OCs in conditional medium with ordinary culture condition (Ctrl), HSC supernatant (M-Ctrl), or conditional medium of etoposide pretreated HSC supernatant (M-DNA-dam), or in the presence of laminin (M-DNA-dam-Laminin) (n=4, * p < 0.05).

Aging is associated with increased oxidative stress and declined DNA repair. However, the response to DNA damage is poorly understood in aged HSCs. We observed that the basal level of γ-H2AX, a marker for DNA double-strand breaks, was higher in aged mice, and DNA damage signals were widespread in livers of aged mice on DDC diet, in contrast to near absence of DNA damage in those of young mice (Figure 5A). We also detected high levels of ROS in aged HSCs (Figure 5B). The increase in the levels of γ-H2AX and senescence-associated markers, p21 and p16, in aged HSCs from DDC-fed mice was confirmed by Western blot (Figure 5C). Meanwhile, in isolated HSCs from aged mice, *Cdkn2a* and *Cdkn1a* were also upregulated (Supplementary Figure S6A). We further explored the relationship between DNA damage and HSC function by treating an immortalized murine HSC cell line, JS1 cells, with etoposide, a DNA damaging agent that has previously been reported to induce DNA double-strand breaks (DSBs) [18]. There was no change in cellular morphology but etoposide-pretreated JS1 cells proliferated slower than control (Supplementary Figure S6B, C). The expression of laminin and α-SMA in JS1 cells was decreased in a dose-dependent manner (Figure 5D). We also found that the proliferation was inhibited in etoposide treated JS1 cells (Figure 5E). We further tested the effect of etoposide-treated HSCs on OCs. JS1 cells were pretreated with low dose of etoposide for 24 hours and then the drug was washed off. After 24 hours, the supernatant, which contained components produced by JS1 cells, was collected, served as HSC-conditional medium. Measurement of laminins in this conditional medium by ELISA indicated that laminins were rich in the HSC cell culture medium and but were decreased with etoposide treatment (Figure 5F). While conditioned medium by control cells remarkably boosted the proliferation of OCs, the conditioned medium by etoposide-treated HSCs lost this ability, which could be restored by the addition of extrinsic laminin (Figure 5G). Taken together, these results indicate that the OC-supporting function of HSCs, via the production of laminin, can be compromised by DNA damage.

### The OC-supporting function of HSCs relies on DNA-PK

**Figure 6:**
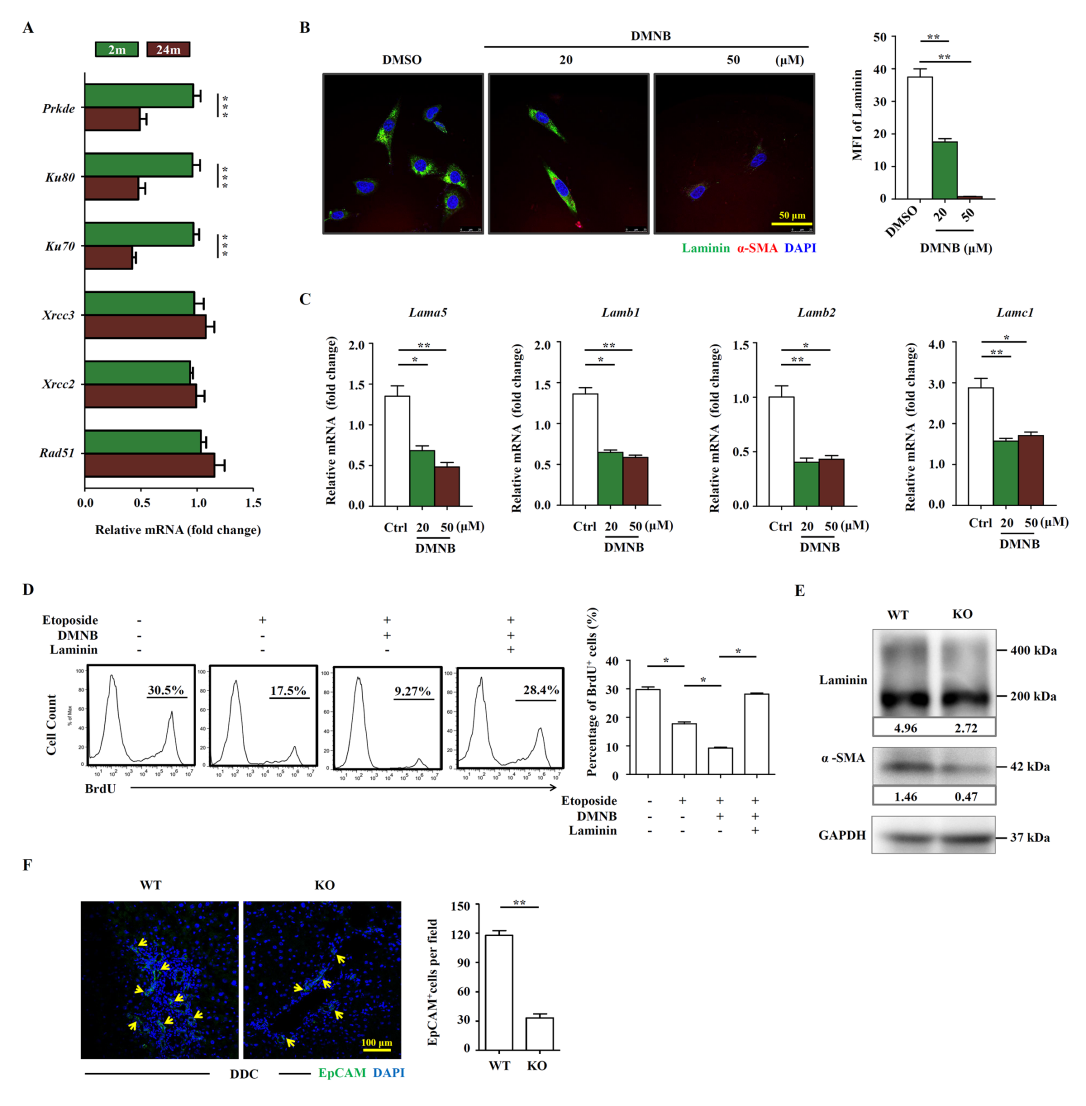
**The OC-supporting function of HSCs relies on DNA-PK pathway.** (**A**) Quantitative Real-time PCR analysis of HR and NEHJ components in HSCs freshly isolated from young (2m) and aged (24m) mice with DDC diet (n=4, *** p < 0.001). (**B**) The expression of α-SMA (red) and laminin (green) in etoposide (0.1 μM) and DMNB treated JS1 cells was detected by confocal. MFI of laminin was quantified (n=5, ** p < 0.01). (**C**) Quantitative Real-time PCR showed that the expression of laminin isoforms in etoposide (0.1 μM) and DMNB treated JS1 cells (n=6, * p < 0.05, ** p <0.01). (**D**) Proliferation of OCs co-cultured with JS1 cells pretreated with etoposide, DMNB or laminin was compared by BrdU analysis. BrdU^+^ cell percentage was quantified (n=4, * p < 0.05). (**E**) Western blot analysis of indicated proteins in HSCs isolated from wild type (WT) and Ku80 knockout (KO) mice fed with DDC. (**F**) Immunofluorescence staining for EpCAM^+^ cells (green) in DDC-fed wild type (WT) versus Ku80 knockout mice (KO). Quantification of EpCAM^+^ cells was shown (n=5, ** p <0.01).

Multiple DNA repair pathways have evolved to deal with various DNA lesions. Homologous recombination (HR) and nonhomologous end joining (NHEJ) are the major pathways for the repair of DSBs. Since the level of DSBs was significantly higher in the liver of aged mice, we investigated whether HR and NHEJ were downregulated in aged liver. We analyzed the expression levels of HR and NHEJ components in HSCs freshly isolated from young and aged mice on DDC diet, respectively. We found that all three components of DNA-PK were expressed at significantly lower levels in aged mice compared to young group, while the HR components remained unchanged in their expression (Figure 6A). 4,5-Dimethoxy-2-nitrobenzaldehyde (DMNB) is a DNA-PK complex inhibitor that specifically inhibits the catalytic activity of DNA-PK [19]. We then subjected JS1 cells to etoposide and DMNB sequentially. Not surprisingly, the cells were impaired in their proliferation in the treatment group (Supplementary Figure S7). Moreover, laminin production was reduced at protein level (Figure 6B) and at mRNA level in JS1 cells treated with DMNB (Figure 6C). The ability of HSCs to promote OC proliferation was significantly reduced in co-culture system after HSCs were pre-treated with etoposide and DMNB (Figure 6D), while addition of laminin could completely restore the proliferation of OCs (Figure 6D). Ku80 is the regulating subunit of DNA-PK complex and mice with a deleted Ku80 gene exhibit premature aging [20]. Then we used Ku80 knockout mice to test the function of DNA-PK in regulating the HSCs’ role on OC activation. Not surprisingly, the laminin production and the HSC activation were decreased in Ku80 knockout mice compared to the wildtype (Figure 6E). In addition, the OC activation by Ku80 null HSCs was also compromised when compared to wildtype HSCs (Figure 6F). Taken together, the results indicate that genome integrity and DNA-PK are essential for the production of laminin by HSCs.

## DISCUSSION

Aging is a systemic process that is associated with chronic low-grade inflammation, stem/progenitor cell dysfunction and cellular senescence, among other features. With aging, senescent tissue-specific stem cells accumulate in various tissues and at sites of pathogenesis in many chronic diseases. However, whether senescent liver progenitor cells accumulate in liver and whether they contribute to age-related pathological changes are not well known. In this study, we found that the activation of OCs in response to liver injury is decreased in aged mice. We also demonstrate that the decline in the activation of OCs to liver injury in aged mice is mainly due to dysfunction of the niche. HSCs participate in the maintenance of healthy OC niche by producing laminin, which promotes the proliferation of OCs via interacting with α6 integrins. In aged mice, the DSBs are repaired less efficiently in HSCs. Induction of DSBs and inhibition of DNA-PK in HSCs result in reduced production of laminin. Our findings provide a novel insight into the mechanisms by which HSCs contribute to functional maintenance of OCs in aged mice.

The functions of tissue stem/progenitor cells usually decline with aging, which is believed to contribute to organism dysfunction. For example, the number of hemopoietic stem cells declines with aging and aged stem cells are prone to differentiate towards myeloid lineage [21,22]. The aging of hemopoietic stem cells might be the reason underlying the observed moderate anemia in the elderly. In satellite cells, which are muscle stem cells, cell-intrinsic factors, such as mitochondrial dysfunction [23], could be responsible for stem cell aging. However, whether liver progenitor cells become senescent and accumulate within liver in contribution to age-related pathological changes is not well known. In this study, while we found no evidence for senescent OCs, but did show that HSCs can become senescent and thus were less supportive for the OCs during liver injury in aged mice.

It is well-known that niche is required for the maintenance of stem/progenitor cell survival and functionality. Some studies indicate that the dysregulation of niche related with aging may cause the decline in tissue regeneration and the corresponding function. Ambrosi *et al* found that aging could induce ectopic adipocyte accumulation in bone marrow cavities, which impairs osteogenesis and hematopoiesis [24]. Heterochronic parabiosis and restoring the levels of IGF-1, GH, Wnt3, TGF-β or GDF11 in aged mice could improve neurogenesis [25]. Meanwhile, we previously reported that aging-associated oxidative stress could change the microenvironment and inhibit liver progenitor cell activation in mice [26].

It is worth mentioning that the debate about whether stem cell itself or niche senescence in aged mice has never stopped. Even it is believed that both cell extrinsic and intrinsic factors may regulate the function of adult stem cells, however, whether and how the cell extrinsic and intrinsic factors interact in ultimately determining tissue homeostasis and repair during aging is less understood [27]. We herein showed that the decline in the activation of OCs in response to liver injury in aged mice is mainly due to the niche dysfunction. This finding is consistent with previous reports of liver progenitor cells being influenced by niche [28,29]. Remodeling of ECM within niche was shown to be required for the hepatic progenitor cell response [30]. ECM components, such as laminin and fibronectin, could induce cultured OCs to differentiate towards β-cell and islet development [31].

ECM is an essential niche component. We found that with aging, the expression of laminin, one of the ECM components, declines within the OC niche. We here showed for the first time that laminin could promote the proliferation of OCs, in the meantime, we found that HSCs could participate in the remodeling of OC niche by producing laminin. On the other hand, HSC secreted hepatocyte growth factor can affect hepatic progenitor cell proliferation, migration and differentiation [11,32]. Pharmaceutical prevention of HSC activation causes a decreased OC response in rats, showing the supportive roles of stromal cells on OCs. Due to the discovery that HSCs regulate OC proliferation via the production of laminin, it is likely that HSCs may also support the OCs by providing a suitable microenvironment in those situations. This finding may help develop new interventional measures for some liver diseases.

It is worth noting that over the past few years, many studies focused on finding the strategies to induce the senescence of HSCs, thus alleviating the liver fibrosis, which is a dynamic process characterized by the excessive accumulation of ECM resulting from many chronic liver injuries of any aetiology, including viral infection, alcoholic liver disease and non-alcoholic steatohepatitis. In this study, we found that the HSCs in aged mice displayed senescent phenotype, and were less activated, together with decreased production of laminin, which showed the negative impact on liver repair in aged mice. Since it is widely recognized that aging is associated with widespread impairment of cellular function and resulting in the increasing risk of many diseases, meanwhile the biological characteristics of senescent HSCs are complicated, better understand of the mechanism of their decreased activation and ECM production may benefit for some definite liver diseases intervention.

In this study, we further found that DSBs accumulated in aged HSCs and the production of laminin was reduced in aged mice under liver injury. DSBs are mainly repaired by two pathways: HR and NHEJ. In vertebrates, NHEJ is regarded as the predominant mechanism for DSBs repair. DNA-PK complex (DNA-PK catalytic subunit, the Ku80/Ku70 heterodimer) and the DNA Ligase IV/XRCC4/XLF complex are central to NHEJ. Impairment in the repair of DSBs will accelerate cellular senescence. We observed that the expression levels of DNA-PK components were downregulated in aged HSCs. Importantly, HSCs treated with DNA-PK inhibitor or etoposide also exhibited decreased expression of laminin. These results suggest that persistence of DSBs may drive the downregulation of laminin. However, the precise mechanism needs to be further investigated.

In summary, OC activation in response to liver injury is greatly compromised in aged mice. The impairment in OC activation is mainly attributed to the senescence of HSCs. The production of laminin that is critical for OC proliferation and function, is downregulated in aged HSCs. Our findings suggest that increasing the supply of laminin in OC niche may help improve the outcomes of chronic liver injury in aged subjects.

## MATERIALS AND METHODS

### Mice

Male C57BL/6 mice and GFP transgenic C57BL/6 mice (6-8 weeks old) were obtained from the Shanghai Laboratory Animal Center at the Chinese Academy of Sciences (Shanghai, China). Ku80 knockout mice, which were originally described by Nussenzweig *et al* [33], were from Professor Changshun Shao in Shandong University. 8-10 weeks old mice were used in this study. All mice were maintained under specific pathogen-free conditions in the animal center of Shanghai Jiao Tong University School of Medicine (Shanghai, China) for up to 2 years. Mice used at 2 and 24 months of age were considered as young or aged mice accordingly [34]. All animal experiments were performed in accordance with the guidelines from the Biomedical Research Ethics Committee of Shanghai Jiao Tong University School of Medicine. All efforts were made to minimize the suffering of mice during experiments.

### Primary OC purification and culture

Mice were fed with 0.1% DDC (Sigma-Aldrich, St. Louis, MO) for 3 weeks to induce the activation of OCs. OCs were isolated as we previously reported [26]. Briefly, livers were perfused from portal vein with collagenase and pronase digestion. Liver cells were resuspended and centrifuged for collection of NPCs. NPCs were centrifuged through a discontinuous gradient of 20% and 50% Percoll^TM^ (Amersham Biosciences, Pittsburgh, PA). Finally, OCs were isolated from these cells fragment using a MiniMACS column containing anti-EpCAM antibody (Miltenyl Biotec, Auburn, CA). The EpCAM^+^ cells were tested for OC markers by flow cytometry and determined to contain OCs at approximately 95% purity. Cells were cultured on type I collagen coated dishes (BD Biosciences, San Jose, CA) in complete medium. Single clones were isolated and expanded *in vitro*.

### HSC purification and culture

Primary HSCs were isolated as described [35]. Briefly, livers were perfused from portal vein with collagenase and pronase digestion, and further digested with enzymes (1 mg/mL collagenase type IV, 1 mg/mL pronase and 2 mg/mL DNase I) and then filtered through 70 μm cell strainers. Cell suspension was centrifuged through a discontinuous gradient with Nycodenz (Axis-Shield, Oslo, Norway) solution and the interphase containing the HSCs was harvested. Cells were transferred into tissue culture flasks with a concentration of 2×10^4^ cells/cm^2^. After incubation for 2 hours, the medium was changed and HSCs were identified by autofluorescence excited by UV.

### Parabiosis model

Parabiosis between a young male and an aged male mouse was constructed by subjecting the mice to full muscle relaxation by intraperitoneal injection with ketamine HCl and xylazine. The corresponding lateral aspects of the two partners were shaved, matching skin incisions were made from the olecranon to the knee joint in both mice, and their subcutaneous fasciae were bluntly dissected to create about 0.5 cm of free skin. An incision was made in the peritoneum of each mouse, and both peritonea were tied with 4-0 vicryl sutures. The dorsal and ventral skins were approximated by continuous silk sutures.

### Cell culture

Primary OCs were cultured in complete medium containing DMEM/F12 1:1 medium, 10% fetal bovine serum (FBS) (Thermo Fisher Scientific, Waltham, MA), 10 ng/mL HGF (PeproTech, Rocky Hill, NJ), 20 ng/mL EGF (PeproTech), 20 ng/mL bFGF (PeproTech), Insulin-Transferrin-Selenium-Ethanolamine (ITS-X, Thermo Fisher Scientific), 50 μg/mL Dexamethasone (Sigma-Aldrich, St. Louis, MO), 20 μM rho-associated protein kinase inhibitor-Y27632 (Sigma-Aldrich), 1% vol/vol Penicillin-Streptomycin (Thermo Fisher Scientific). Primary HSCs were cultured in DMEM (Thermo Fisher Scientific) supplemented with 10% heat-inactivated FBS, 1% Penicillin/Streptomycin, 2 mM L-Glutamine (Thermo Fisher Scientific), 1 mM sodium pyruvate (Thermo Fisher Scientific), and 10 mM HEPES (Thermo Fisher Scientific). The immortalized murine HSC cell line, JS1 cell, was kindly provided by Professor Scott L. Friedman (Department of Medicine, Mount Sinai School of Medicine, New York) and Dr. Jinsheng Guo (Zhongshan Hospital, Shanghai Medical College, Fudan University, Shanghai, China). JS1 cells were cultured with DMEM supplemented with 10% heat-inactivated FBS, 1% Penicillin/Streptomycin, 2 m L-Glutamine, 1 mM sodium pyruvate, and 10 mM HEPES. Etoposide was purchased from Sigma-Aldrich. DMNB was purchased from Millipore. Laminin was purchased from Sigma-Aldrich.

### Isolation of liver extract

The liver tissues were cut and put into PBS, 1g tissue with 9 mL PBS. Then, the homogenate extraction was centrifuged (5000 g, 15 min). The supernatant was used as liver extract.

### Immunofluorescence

O.C.T. Compound (SAKURA Tissue-Tek^®^, Torrance, CA) embedded frozen liver tissues were cut into 5 μm sections. After washed with PBS, sections were fixed with paraformaldehyde, penetrated with Triton X-100 and blocked with bovine serum albumin, primary antibodies anti-α-SMA, anti-EpCAM, anti-laminin, anti-γ-H2AX (all from AbCAM, Cambridge, UK) were probed overnight at 4 °C, proper Alexa Flour conjugated secondary antibodies (Thermo Fisher Scientific) were used to detect the target protein. Images were collected by confocal microscopy (Zeiss LSM710, Oberkochen, Germany).

### Cell proliferation assay

Briefly, cells were seeded in 96-well cell culture plate at a density of 1×10 ^4^ cells per well, then incubated for 24 hours. Then the medium was removed and the cells were treated with new FBS free medium followed by the addition of CCK-8 solution (Dojindo Laboratories, Mashikimachi, kamimashiki gun Kumamoto, Japan). After 2 hours, the BioTEK (Winooski, VT) was used to record the absorbance at 450 nm. In some experiments, the cytokines/growth factors were added into the OC culture medium for 24 hours with different doses. The factors are IGF-1 (R&D Systems, Minneapolis, USA, 791-MG-050), GH (ProSpec, Rehovot, Israel, CYT-540), Wnt3a (R&D Systems, Minneapolis, USA, 1324-WN-010), TGF-β1 (R&D Systems, Minneapolis, USA, 7666-MB-005) and GDF11(R&D Systems, Minneapolis, USA, 1958-GD-010).

### Co-culture of OCs with HSCs and BrdU analysis

Primary HSCs were isolated from naive GFP transgenic mice if needed. GFP-HSCs were isolated and cultured in 6-well plates for 6 days for self-activation, and then OCs were seeded into the plate at a ratio of 1:5 (OCs versus HSCs) with half of each medium. After 24 hours, BrdU was incorporated into cells in the co-culture system with a concentration of 100 μM 4 hours before analysis, and then operated according to the instruction of BD Pharmingen BrdU Flow Kits (BD Biosciences, San Jose, CA). In some experiments, JS1 cells were used to substitute HSCs since primary isolated HSCs can’t be passaged well.

Transwell co-culture system was induced in some condition. Basically, JS1 cells were seeded into the 6-well plates with 1×10 ^5^ per well, after 24 hours, a 24 mm diameter and 0.4 μm pore size transwell (Merck Millipore, Darmstadt, Germany) is fixed into the 6-well plates. Then OCs were seeded into the transwell with 5×10 ^4^ per well. After 24 hours co-culture, BrdU was incorporated into cells in the co-culture system with a concentration of 100 μM for 4 hours before analysis, and then operated according to the instruction of BD Pharmingen BrdU Flow Kits.

### Western blot

Liver tissue or cell extract with RIPA Lysis and Extraction Buffer (Thermo Fisher Scientific). Then, total protein was resolved by SDS-PAGE and immunoblots probed overnight at 4 °C with primary antibody anti -GAPDH (Cell Signaling Technology, Danvers, MA), anti-p53 (Cell Signaling Technology), anti-p21 (Santa Cruz, Dallas, Texas), anti-p16 (Santa Cruz), anti-α-SMA (Abcam), anti-laminin (Abcam), and appropriate horseradish peroxidase-labelled secondary antibodies (Cell Signaling Technology). The immunoreactive bands were visualized with ECL reagents (Merck Millipore) and the signal was collected with GE image Quant LAS 4010 (General Electric Company, Fairfield, Lowa), for some immunoblots, the band densitometry was quantified using ImageJ software (National Institutes of Health). To analyze, each protein is normalized with GAPDH.

### Quantitative Real-time PCR analysis

Liver tissue or cell RNA was prepared using TRIzol (Thermo Fisher Scientific,). An amount of 500 ng RNA was used for 10 μL cDNA synthesis using PrimeScript™ RT Master Mix (Takara Bio Inc., Otsu, Shiga, Japan). Quantitative real-time PCR was performed for cDNA samples using FastStart Universal SYBR Green Master (Rox) (Roche, Basel, Switzerland) and signal was collected by ABI Prism 7900HT (Applied Biosystems, Carlsbad, CA). Primers used are shown in Supplementary Table S1.

### Flow cytometry

Primary isolated OC were cultured in vitro and passaged for about 6-8 times. The OCs were harvested and then incubated with anti-EpCAM-APC, anti-Sca-1-PE, anti-CD44-PE, anti-CD49f-APC, anti-CD45-APC, anti-CD34-PE, anti-c-kit-FITC, anti-α6 integrin-APC, anti-β1 integrin-PercpCy5.5, anti-β4 integrin-PercpCy5.5 (all from ebioscience, San Diego, CA) at 4°C for 30 mins, after that, cells were fixed with 1% paraformaldehyde and analyzed by a BD Calibur (Becton, Dickinson and Company, New Jersey) and Flowjo software (Tree Star, San Carlos, CA).

### ROS detection

Assessment of intracellular ROS was assessed using the ROS detection reagents (Thermo Fisher Scientific). In brief, 1 × 10^4^ cells were harvested and cultured under the same conditions described earlier. The cells were then incubated with DCF-DA (2,7-dichlorofluorescin diacetate) (10 μM) for 30 mins at room temperature to detect ROS.

### Statistical analysis

Differences were evaluated using Statistical Package for Social Science software (version 16.0, SPSS Inc., Chicago, IL). Differences between two groups were compared using unpaired Student *t* test or Mann-Whitney test. Multiple treatment groups were compared by one-way ANOVA followed by Dunnett post-hoc tests or two-way ANOVA followed by Bofferoni post-hoc tests to compare difference between each two groups, respectively. All data were presented as mean ± SEM. Significance was expressed as * p < 0.05, ** p < 0.01, and *** p < 0.001.

## SUPPLEMENTARY MATERIAL

Supplementary Figure S1

Supplementary Figure S2

Supplementary Figure S3

Supplementary Figure S4

Supplementary Figure S5

Supplementary Figure S6

Supplementary Figure S7

Supplementary Table S1
