# Supplementary material for: Reduced production of laminin by hepatic stellate cells contributes to impairment in oval cell response to liver injury in aged mice
Source: Aging (Albany NY). 2018 Dec 4;10(12):3713–35. doi: 10.18632/aging.101665 (PMC6326669; doi:10.18632/aging.101665)
Supplement: Supplementary Figure S1 [file aging-10-101665-s001.pdf]

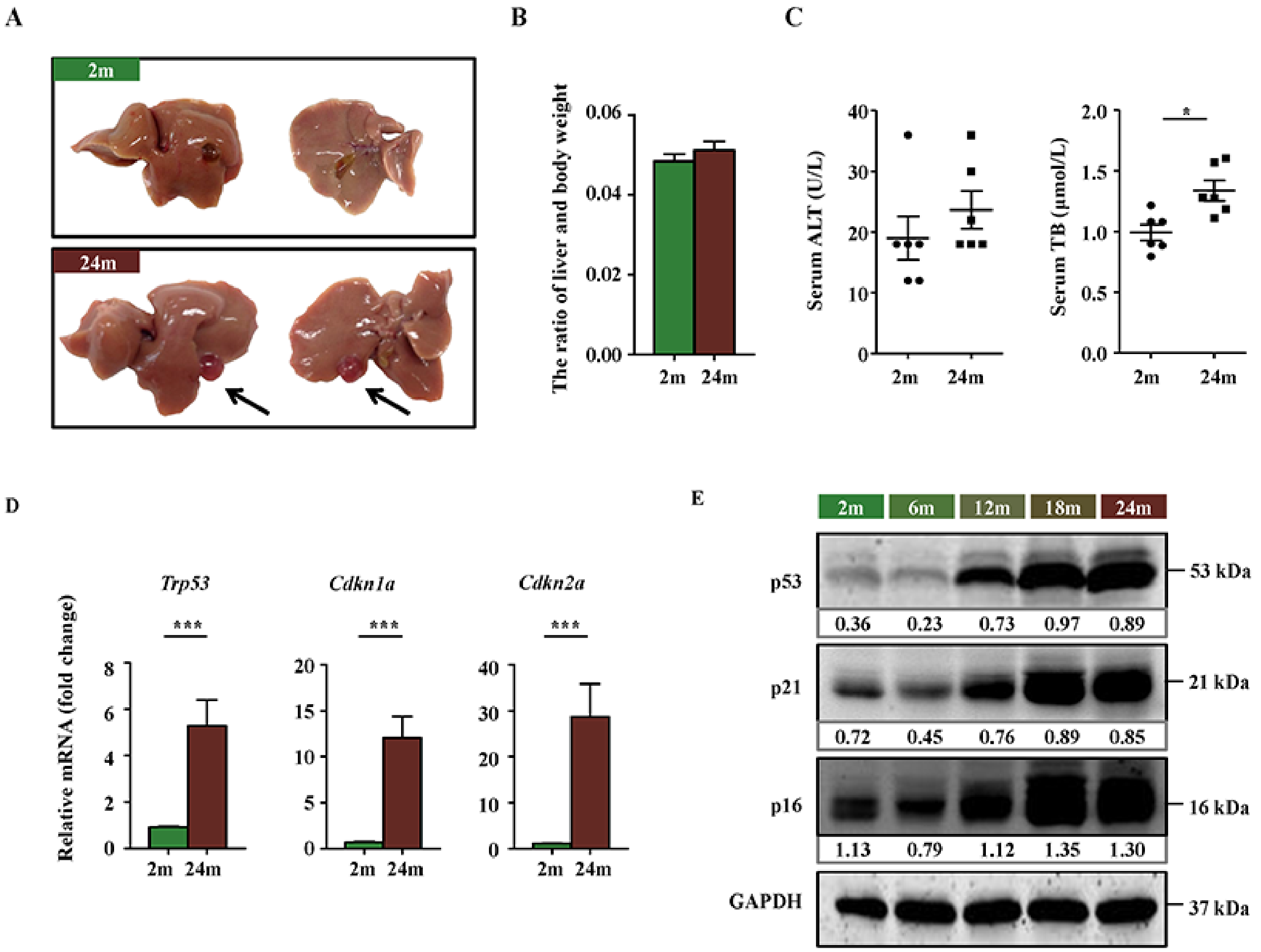

Supplementary Figure S1. The morphology and basic function of liver between young and aged mice. (**A**) The photos of young (2m) and aged (24m) mice liver. (**B**) The whole liver and the body were weighted. The ratio of liver and body weight was calculated (n=3). (**C**) Serum alanine aminotransferase (ALT) and total bilirubin (TB) of young (2m) and aged (24m) mice was tested by ELISA (n=6, * $p < 0.05$). (**D**) Quantitative Real-time PCR showed the expression levels of *Trp53*, *Cdkn1a* and *Cdkn2a* between 2m and 24m liver tissues (n=9, *** $p < 0.001$). (**E**) Western blot showed the expression of p53, p21 and p16 in 2m, 6m, 12m, 18m and 24m liver tissues.
