# Supplementary material for: Reduced production of laminin by hepatic stellate cells contributes to impairment in oval cell response to liver injury in aged mice
Source: Aging (Albany NY). 2018 Dec 4;10(12):3713–35. doi: 10.18632/aging.101665 (PMC6326669; doi:10.18632/aging.101665)
Supplement: Supplementary Figure S2 [file aging-10-101665-s002.pdf]

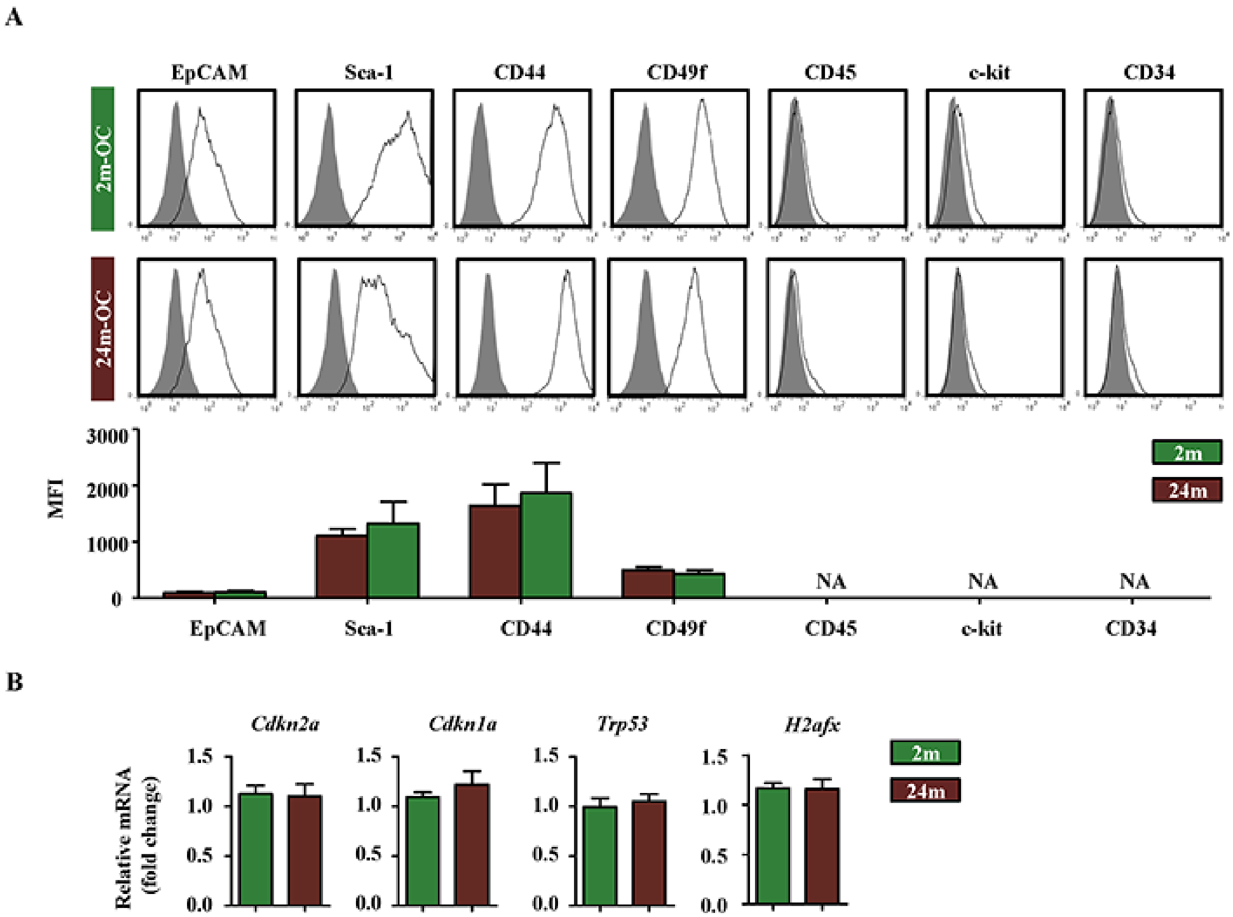

Supplementary Figure S2. The phenotype of OCs. (**A**) Flow cytometry analysis showed the surface marker of OCs between young (2m-OC) and aged mice (24m-OC). The quantification of mean fluorescence intensity (MFI) of OC markers was calculated (n=3). (**B**) Quantitative Real-time PCR showed the xpression levels of Cdkn2a, Cdkn1a, Trp53 and h2afx in primary OCs isolated from the young (2m) and aged (24m) mice with DDC diet (n=5).
