# Supplementary material for: Reduced production of laminin by hepatic stellate cells contributes to impairment in oval cell response to liver injury in aged mice
Source: Aging (Albany NY). 2018 Dec 4;10(12):3713–35. doi: 10.18632/aging.101665 (PMC6326669; doi:10.18632/aging.101665)
Supplement: Supplementary Figure S3 [file aging-10-101665-s003.pdf]

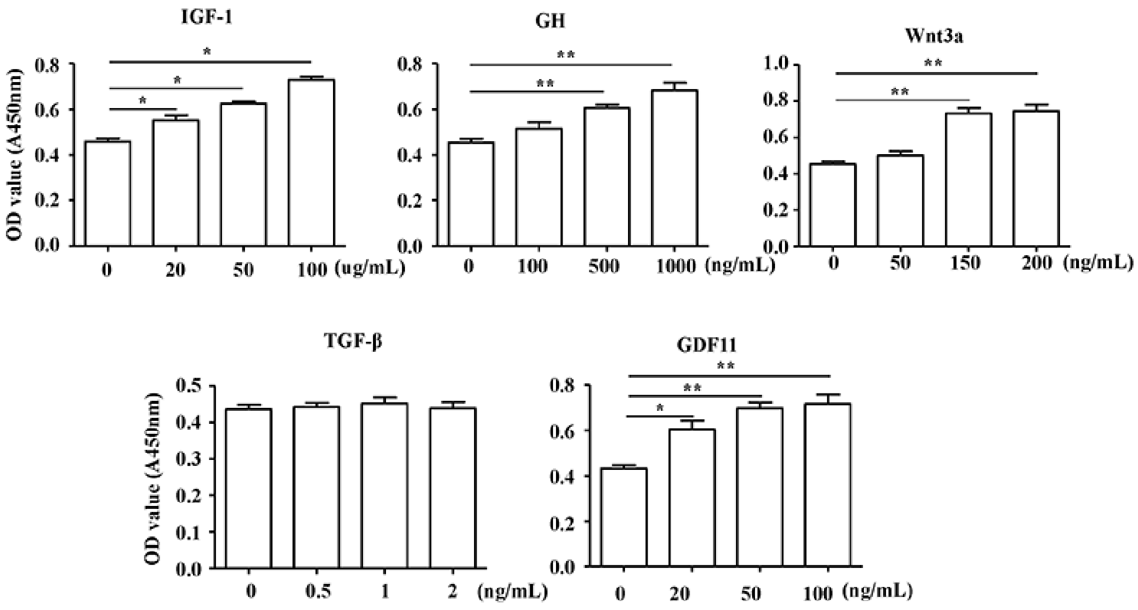

Supplementary Figure S3. The proliferation of OCs under the treatment of several cytokines. OCs were treated with IGF-1 (0, 20, 50, 100 ug/ml), GH (0, 100, 500, 1000 ng/ml), Wnt3a (0, 50, 150, 200 ng/ml), TGF-β (0, 0.5, 1, 2 ng/ml) and GDF11 (0,20, 50, 100 ng/ml) for 24 hours, and the CCK-8 test was performed (n=5, * $p< 0.05$, ** $p< 0.01$).
