# Supplementary material for: Reduced production of laminin by hepatic stellate cells contributes to impairment in oval cell response to liver injury in aged mice
Source: Aging (Albany NY). 2018 Dec 4;10(12):3713–35. doi: 10.18632/aging.101665 (PMC6326669; doi:10.18632/aging.101665)
Supplement: Supplementary Figure S4 [file aging-10-101665-s004.pdf]

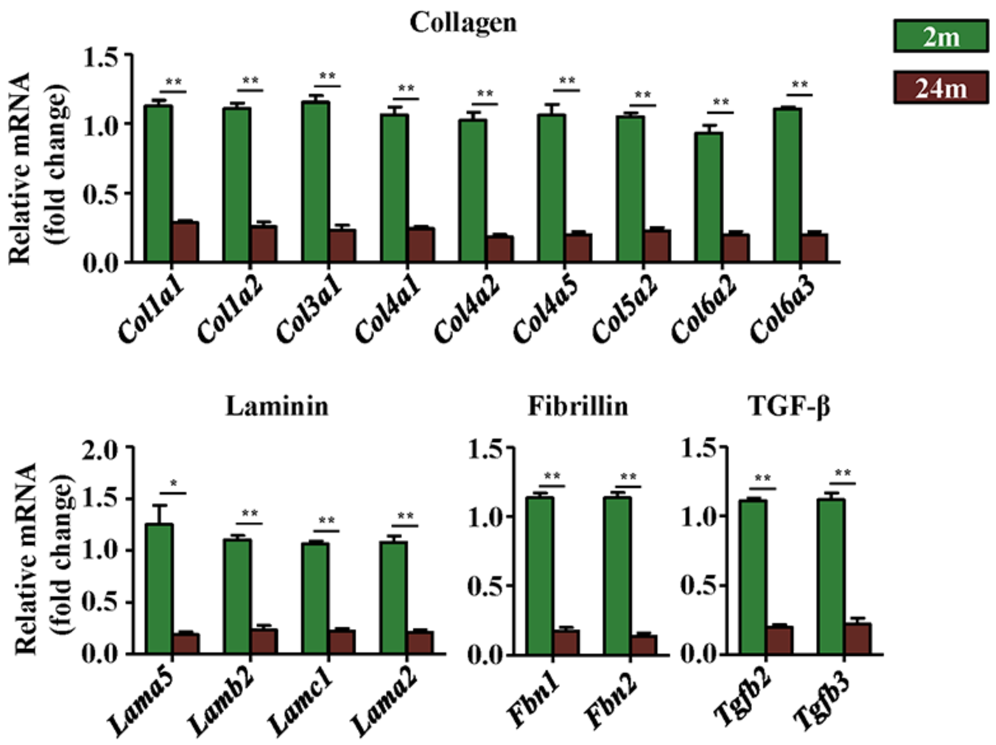

Supplementary Figure S4. The expression levels of ECM-related genes in the liver tissues of young and aged DDC mice. The expression levels of collagen isoform genes (Col1a1, Col1a2, Col3a1, Col4a1, Col4a2, Col4a5, Col5a2, Col6a2 and Col6a3), laminin isoform genes (Lama2, Lama5, Lamb2 and Lamc1), fibrillin isoforms (Fbn1 and Fbn2) and TGF-β isoform genes (Tgfb2 and Tgfb3) were quantified by quantitative Real-time PCR (n=5, * $p < 0.05$, ** $p < 0.01$).
