# Supplementary material for: Reduced production of laminin by hepatic stellate cells contributes to impairment in oval cell response to liver injury in aged mice
Source: Aging (Albany NY). 2018 Dec 4;10(12):3713–35. doi: 10.18632/aging.101665 (PMC6326669; doi:10.18632/aging.101665)
Supplement: Supplementary Figure S5 [file aging-10-101665-s005.pdf]

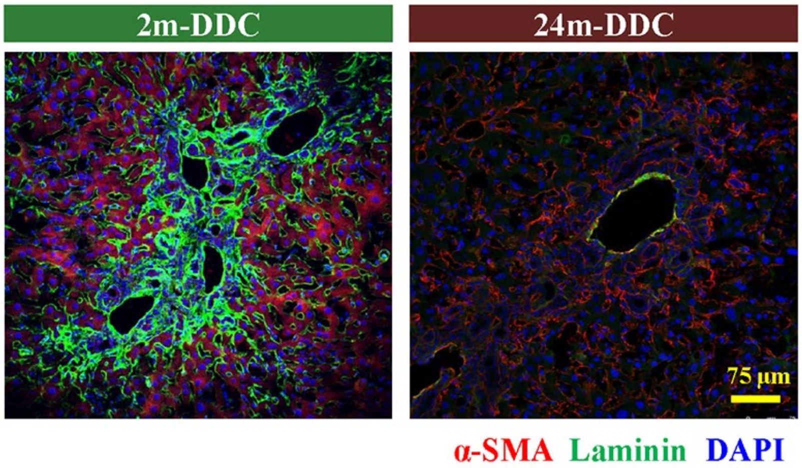

Supplementary Figure S5. HSCs produced laminin within the liver under DDC diet. Immunofluorescence staining for α-SMA+ (red) and laminin+ (green) cells young (2m) and aged (24m) mice with DDC diet (Scare bar=75 µm).
