# Supplementary material for: Reduced production of laminin by hepatic stellate cells contributes to impairment in oval cell response to liver injury in aged mice
Source: Aging (Albany NY). 2018 Dec 4;10(12):3713–35. doi: 10.18632/aging.101665 (PMC6326669; doi:10.18632/aging.101665)
Supplement: Supplementary Figure S6 [file aging-10-101665-s006.pdf]

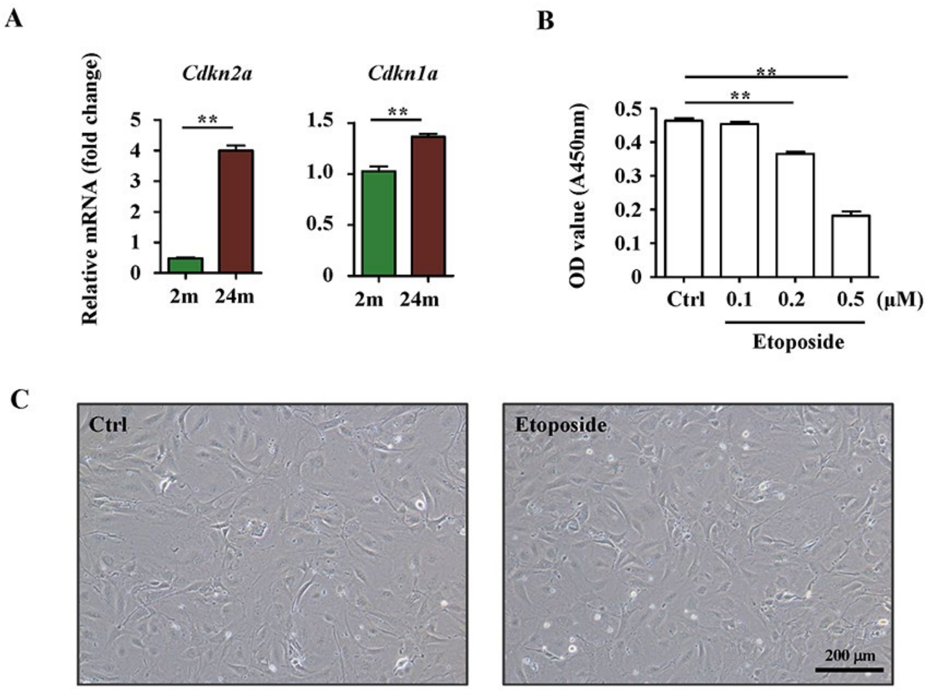

Supplementary Figure S6. The expression level of proliferation associated genes in HSCs and the biological characteristics of JS1 cells in the condition of slightly DNA damage. (**A**) The expression levels of *Cdkn2a* and *Cdkn1a* in primary HSCs isolated from the young (2m) and aged (24m) mice with DDC diet was quantified by quantitative Real-time PCR (n=6, ** p < 0.01). (**B**) JS1 cells were pretreated with 0.1 μM, 0.2 μM or 0.5 μM etoposide for 24 hours, then washed with fresh medium and continue to culture for 24 hours, the supernatant was collected as OC conditional medium. OCs were cultured in the conditional medium for 24 hours, the CCK-8 test was performed (n=6, ** p< 0.01). (**C**) JS1 cells were treated with 0.5 μM etoposide for 24 hours, the morphology of these cells was shown (Scare bar=200 μm).
