# Supplementary material for: Reduced production of laminin by hepatic stellate cells contributes to impairment in oval cell response to liver injury in aged mice
Source: Aging (Albany NY). 2018 Dec 4;10(12):3713–35. doi: 10.18632/aging.101665 (PMC6326669; doi:10.18632/aging.101665)
Supplement: Supplementary Figure S7 [file aging-10-101665-s007.pdf]

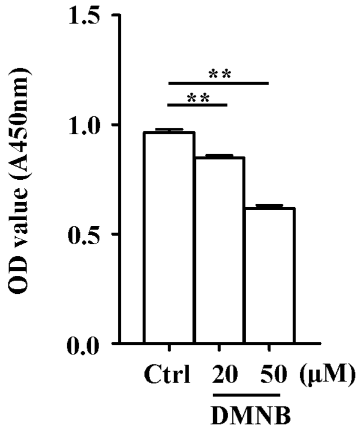

Supplementary Figure S7. The proliferation rate of JS1 cells treated with DMNB. JS1 cells were pretreated with low dose of etoposide (0.1 μM). Then DMNB was added into the medium (20 μM and 50 μM, DMSO was added as control), the JS1 cells proliferation rate was measured by CCK-8 test (n=6, ** $p < 0.01$).
