# Supplementary material for: Reduced production of laminin by hepatic stellate cells contributes to impairment in oval cell response to liver injury in aged mice
Source: Aging (Albany NY). 2018 Dec 4;10(12):3713–35. doi: 10.18632/aging.101665 (PMC6326669; doi:10.18632/aging.101665)
Supplement: Supplementary Table S1 [file aging-10-101665-s008.docx]

**Supplementary Table S1. Primers used in the manuscript.**

| **In the main text** | |
| --- | --- |
| ***Acta2*** | Forward: 5’-CTGACAGAGGCACCACTGAA-3’ |
|  | Reverse: 5’-CATCTCCAGAGTCCAGCACA-3’ |
| ***Afp*** | Forward: 5’-TCACATCCACGAGGAGTGTTG-3’ |
|  | Reverse: 5’-GCGTGAATTATGCAGAAGCCTA-3’ |
| ***Ccnd1*** | Forward: 5’-TGACTGCCGAGAAGTTGTGC-3’ |
|  | Reverse: 5’-GGAGGGTGGGTTGGAAATG-3’ |
| ***Epcam*** | Forward: 5’-GGAGTCCCTGTTCCATTCTTCT-3’ |
|  | Reverse: 5’-GCGATGACTGCTAATGACACCA-3’ |
| ***Gapdh*** | Forward: 5’-AATGGATTTGGACGCATTGGT-3’ |
|  | Reverse: 5’-TTTGCACTGGTACGTGTTGAT-3’ |
| ***Ku70*** | Forward: 5’-GGAGTCAAGCAAGCTGGAAGA-3’ |
|  | Reverse: 5’-AGAACTCGCTTTTTGGTCTCCTT-3’ |
| ***Ku80*** | Forward: 5’-GACTTGCGGCAATACATGTTTTC-3’ |
|  | Reverse: 5’-AAGCTCATGGAATCAATCAGATCA-3’ |
| ***Lama5*** | Forward: 5’-CGCAGGTATTATTACAGCATCAAAG-3’ |
|  | Reverse: 5’-CTGGACAGCATCGGTCACAAG-3’ |
| ***Lamb1*** | Forward: 5’-ATGTGACTCCCGAGACCCTTA-3’ |
|  | Reverse: 5’-ACACCATTTTCCGATTGCCAC-3’ |
| ***Lamb2*** | Forward: 5’-GAACTTCGCTTGGGCCTACTT-3’ |
|  | Reverse: 5’-GGTGGCTGGATAGCAGCTT-3’ |
| ***Lamc1*** | Forward: 5’-TGCCGGAGTTTGTTAATGCC-3’ |
|  | Reverse: 5’-TGGTTGTTGTAGTCGGTCAGG-3’ |
| ***Prkdc*** | Forward: 5’-GCCCATGAGCTTAGGTTTCAAT-3’ |
|  | Reverse: 5’-CTAAGAGCTTTCAGCAGGTTCACA-3’ |
| ***Prom1*** | Forward: 5’-AACACACACATAGTCAGACGGG-3’ |
|  | Reverse: 5’-CCGGTTATGAATTACGTTCCCA-3’ |
| ***Rad51*** | Forward: 5’-AAGTTTTGGTCCACAGCCTATTT-3’ |
|  | Reverse: 5’-CGGTGCATAAGCAACAGCC-3’ |
| ***Xrcc2*** | Forward: 5’-GGAAAGGCCCACATGTGAGT-3’ |
|  | Reverse: 5’-GGATCGTTTGTGACATAGGCATT-3’ |
| ***Xrcc3*** | Forward: 5’-CCTGAGGAGCTGATCGAGAAGA-3’ |
|  | Reverse: 5’-CGGCCGCGTGTTCAAT-3’ |
| **In the supporting text** | |
| ***Cdkn2a*** | Forward: 5’-CGTTCACGTAGCAGCTCTTC-3’ |
|  | Reverse: 5’-GCACGATGTCTTGATGTCCC-3’ |
| ***Cdkn1a*** | Forward: 5’-CCTGGTTCCTTGCCACTTCTT-3’ |
|  | Reverse: 5’-CTGTTCTAGGCTGTGACTGCTTC-3’ |
|  |  |
| ***Gapdh*** | Forward: 5’-AATGGATTTGGACGCATTGGT-3’ |
|  | Reverse: 5’-TTTGCACTGGTACGTGTTGAT-3’ |
| ***Trp53*** | Forward: 5’-GCCGACCTATCCTTACCATCA-3’ |
|  | Reverse: 5’-GGCAGGCACAAACACGAAC-3’ |

|  |  |
| --- | --- |
| ***H2afx*** | Forward: 5’-GGCCTCCAGTTCCCAGTG-3’ |
|  | Reverse: 5’-TCAGCGGTGAGGTACTCCAG-3’ |
| ***Lama5*** | Forward: 5’-CGCAGGTATTATTACAGCATCAAAG-3’ |
|  | Reverse: 5’-CTGGACAGCATCGGTCACAAG-3’ |
| ***Lama2*** | Forward: 5’- ACCAGAGGCACCCGATTAC-3’ |
|  | Reverse: 5’- GGGTTTGTACTCCACGTCATC-3’ |
| ***Lamb2*** | Forward: 5’-GAACTTCGCTTGGGCCTACTT-3’ |
|  | Reverse: 5’-GGTGGCTGGATAGCAGCTT-3’ |
| ***Lamc1*** | Forward: 5’-TGCCGGAGTTTGTTAATGCC-3’ |
|  | Reverse: 5’-TGGTTGTTGTAGTCGGTCAGG-3’ |
| ***Col1a1*** | Forward: 5’-TAAGGGTCCCCAATGGTGAGA-3’ |
|  | Reverse: 5’-GGGTCCCTCGACTCCTACAT-3’ |
| ***Col1a2*** | Forward: 5’-GGTGAGCCTGGTCAAACGG-3’ |
|  | Reverse: 5’-ACTGTGTCCTTTCACGCCTTT-3’ |
| ***Col3a1*** | Forward: 5’-CCTGGCTCAAATGGCTCAC-3’ |
|  | Reverse: 5’-GACCTCGTGTTCCGGGTAT-3’ |
| ***Col4a1*** | Forward: 5’-CCTGGCACAAAAGGGACGA-3’ |
|  | Reverse: 5’-ACGTGGCCGAGAATTTCACC-3’ |
| ***Col4a2*** | Forward: 5’-GACCGAGTGCGGTTCAAAG-3’ |
|  | Reverse: 5’-CGCAGGGCACATCCAACTT-3’ |
| ***Col4a5*** | Forward: 5’-TTCCAGGTTTAGAAGGTCATCCA-3’ |
|  | Reverse: 5’-ACGTTCTCCCTTGGTTCCATT-3’ |
| ***Col5a2*** | Forward: 5’-ACAGGTGAAGTGGGATTCTCA-3’ |
|  | Reverse: 5’-CCATAGCACCCATTGGACCA-3’ |
| ***Col6a2*** | Forward: 5’-CCAGTCAACGTGTATTTCGTGT-3’ |
|  | Reverse: 5’-TAGGCTCTTAGTGAAGGAGGC-3’ |
| ***Col6a3*** | Forward: 5’-CCGCATCGAGGATAACGTG-3’ |
|  | Reverse: 5’-ATGCAGGCGACAGCACAAT-3’ |
| ***Fbn1*** | Forward: 5’-TGTGGGGATGGATTCTGCTC-3’ |
|  | Reverse: 5’-AGTGCCGATGTACCCTTTCTG-3’ |
| ***Fbn2*** | Forward: 5’-GAGCCAAATCAATTCAGCAGTG-3’ |
|  | Reverse: 5’-GGTTGTCCACAGTAAGTTCCGA-3’ |
| ***Tgfb2*** | Forward: 5’-TCGACATGGATCAGTTTATGCG-3’ |
|  | Reverse: 5’-CCCTGGTACTGTTGTAGATGGA-3’ |
| ***Tgfb3*** | Forward: 5’-ATGACCCACGTCCCCTATCAG-3’ |
|  | Reverse: 5’-GCCAGTCCCTGGATCATGT-3’ |
